# Combination of DNA Vaccine and Immune Checkpoint Blockades Improves the Immune Response in an Orthotopic Unresectable Glioblastoma Model

**DOI:** 10.3390/pharmaceutics14051025

**Published:** 2022-05-10

**Authors:** Mathilde Bausart, Kevin Vanvarenberg, Bernard Ucakar, Alessandra Lopes, Gaëlle Vandermeulen, Alessio Malfanti, Véronique Préat

**Affiliations:** Advanced Drug Delivery and Biomaterials, Louvain Drug Research Institute, UCLouvain, 1200 Brussels, Belgium; mathilde.bausart@uclouvain.be (M.B.); kevin.vanvarenberg@uclouvain.be (K.V.); bernard.ucakar@uclouvain.be (B.U.); alessandra.lopes090790@gmail.com (A.L.); gaelle.vandermeulen@gmail.com (G.V.); alessio.malfanti@uclouvain.be (A.M.)

**Keywords:** glioblastoma, plasmid DNA vaccine, immune checkpoint blockade, combination immunotherapy

## Abstract

Combination immunotherapy has emerged as a promising strategy to increase the immune response in glioblastoma (GBM) and overcome the complex immunosuppression occurring in its microenvironment. In this study, we hypothesized that combining DNA vaccines—to stimulate a specific immune response—and dual immune checkpoint blockade (ICB)—to decrease the immunosuppression exerted on T cells—will improve the immune response and the survival in an orthotopic unresectable GL261 model. We first highlighted the influence of the insertion position of a GBM epitope sequence in a plasmid DNA vaccine encoding a vesicular stomatitis virus glycoprotein (VSV-G) (here referred to as pTOP) in the generation of a specific and significant IFN-γ response against the GBM antigen TRP2 by inserting a CD8 epitope sequence in specific permissive sites. Then, we combined the pTOP vaccine with anti-PD-1 and anti-CTLA-4 ICBs. Immune cell analysis revealed an increase in effector T cell to Treg ratios in the spleens and an increase in infiltrated IFN-γ-secreting CD8 T cell frequency in the brains following combination therapy. Even if the survival was not significantly different between dual ICB and combination therapy, we offer a new immunotherapeutic perspective by improving the immune landscape in an orthotopic unresectable GBM model.

## 1. Introduction

Despite an aggressive standard of care (SOC) consisting of maximal surgical resection followed by chemoradiation, the prognosis of patients suffering from glioblastoma (GBM) remains poor, with a median survival lower than 2 years [1,2]. Even with advanced imaging and monitoring methods, total resection of the tumor is almost impossible to achieve due to the microscopic infiltration of GBM cells and/or tumor location; tumor recurrences are, therefore, currently inevitable. The prognosis of patients with unresectable GBM, which counts for approximately 35% of the patients, is even poorer [3,4].

Immunotherapy has revolutionized the field of cancer treatment. Immune checkpoint blockades (ICBs) were firstly approved with ipilimumab—a monoclonal antibody (mAb) inhibiting cytotoxic T-lymphocyte antigen 4 (CTLA-4)—for the treatment of melanoma in 2011 [5]. In 2014, the first anti-programmed cell death protein-1 (PD-1) mAb, nivolumab, was approved for the treatment of metastatic melanoma [6]. Since then, the use of ICBs has been extended to many other cancers. In the past decade, research on therapeutic vaccines has also extended, given their potential to generate specific T-cell responses against tumors [7]. However, challenges remain, and most cancer patients, including GBM patients, still do not respond to current immunotherapies.

Nowadays, there is no FDA-approved immunotherapy for GBM [8]. Moreover, recent phase III clinical trials, including ICBs or therapeutic vaccines (either as monotherapies or in addition to the SOC) for GBM treatment, have failed to improve the median overall survival of patients [9,10,11]. The negligible efficacy of immunotherapy for GBM resides in multiple factors, including (i) the high invasiveness of GBM cells [12], (ii) the presence of the blood–brain barrier (BBB) impeding the efficacy of most systemic treatments [13], (iii) the highly immunosuppressive tumor microenvironment (TME) [14], (iv) the low mutation burden [15], (v) the intra- and inter-tumoral heterogeneity [16,17] and (vi) the lymphopenia induced by SOC and corticotherapy [18]. Given the specific features of GBM and its TME, combining different immunotherapeutic approaches is a promising strategy for overcoming GBM multifactorial immunosuppression and increase antitumor immune response [8].

**Figure 1:**
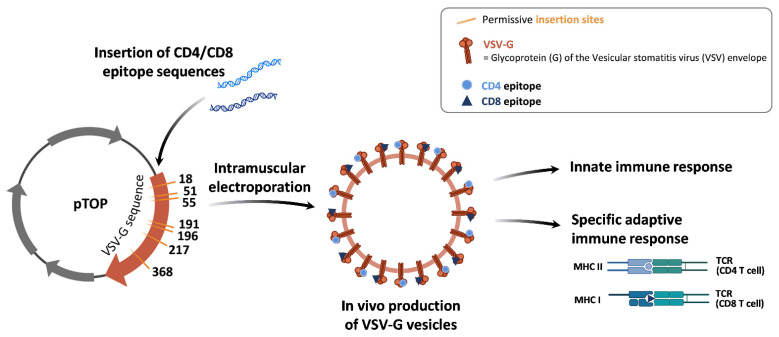
Schematic representation of pTOP vaccine. The pTOP is a plasmid DNA encoding a VSV-G sequence modified by the insertion of tumor epitope sequences in permissive sites. After administration of pTOP, cells will produce VSV-G vesicles incorporating CD4 and/or CD8 epitopes. These immunogenic vesicles will activate both the innate immunity and the adaptive immunity by presentation of the epitopes through the MHC class I or II molecules to the CD8 or CD4 T cells. (TCR = T cell receptor).

We developed pTOP (plasmid to deliver T cell epitopes), a DNA vaccine encoding a vesicular stomatitis virus glycoprotein (VSV-G) engineered by the insertion of tumor epitopes through permissive sites in its sequence (Figure 1) [19]. The VSV-G viral protein is recognized by the immune system and we demonstrated that the administration of a plasmid encoding this protein led to the production of immunogenic VSV-G vesicles and induced the activation of the innate immune system [19]. Electroporation was used to deliver the plasmids in order to increase the delivery and distribution of DNA into cells as well as the gene expression [20]. Injections were performed intramuscularly as we previously showed that the muscle was the best administration site for DNA vaccine electroporation in inducing a strong immune response [21]. We further demonstrated that the insertion of tumor epitopes in specific positions in the VSV-G sequence led to MHC class I and II restricted immune response. Overall, pTOP generated a potent anticancer immune response with stimulation of both innate and adaptive immunity in different cancer models, including GBM when combined with surgical resection [19].

We hypothesized that combining pTOP with ICB could synergize to induce a potent immune response and drive effector T cells in the brain. Indeed, recent studies on brain metastases models demonstrated that the intracranial efficacy of ICBs was occurring only when extracranial tumors were present, by a peripheral expansion of effector T cells activated outside the brain [22,23]. We assumed that the DNA vaccine would induce similar effects by activating a peripheral antigen-specific immune response. Therefore, to check this hypothesis, we selected the most efficient insertion position in the VSV-G sequence to stimulate a specific immune response against a selected CD8 GBM epitope. Indeed, it is known that the position of an epitope in a protein sequence may influence its processing by the proteolysis machinery and therefore impact its binding to MHC class I molecules and presentation to CD8 T cells [24]. Then, we combined the vaccine with anti-PD-1 and anti-CTLA-4 ICBs to decrease immunosuppression.

## 2. Materials and Methods

### 2.1. Plasmids

The pVAX2 plasmid was used as a backbone for the pTOP constructs [19,21]. Further, pTOP refers to plasmids encoding the VSV-G sequence modified by the insertion of tumor epitope sequences in permissive insertion sites. Here, we used the GBM CD8 epitope TRP2_180–188_. The pTOP constructs were generated by Gibson assembly cloning. Codon optimization of the modified VSV-G gene sequences was performed using the GeneOptimizer software algorithm (ThermoFisher Scientific, Waltham, MA, USA). Sequences were ordered as gBlocks™ Gene Fragments (Integrated DNA Technologies, Coralville, IA, USA) and assembled using the NEBuilder^®^ HiFi DNA Assembly Cloning Kit (New England Biolabs, Ipswich, MA, USA). Plasmids were amplified in NEB^®^ 5-alpha Competent *E. coli* (New England Biolabs, Ipswich, MA, USA) and purified using the EndoFree Plasmid Mega Kit (Qiagen, Hilden, Germany). Plasmid sequences were verified by Sanger DNA sequencing (Genewiz, Leipzig, Germany). The seven plasmids obtained are named according to the insertion position of TRP2_180–188_ in the VSV-G protein sequence: pTOP_TRP2(18), pTOP_TRP2(51), pTOP_TRP2(55), pTOP_TRP2(191), pTOP_TRP2(196), pTOP_TRP2(217) and pTOP_TRP2(358). pVAX2 expressing the complete sequence of TRP2 (pVAX2-TRP2) and pVAX2 expressing only the VSV-G sequence (pVAX2-VSVG) were used as controls. The protein sequence of VSV-G with the positions of the permissive insertion sites is available in Figure S1.

### 2.2. GL261 Cell Line

GL261 murine glioma cells were purchased from the DSMZ (German Collection of Microorganisms and Cell Cultures GmbH, Leibniz institute, Germany). They were cultured in DMEM (Gibco™, ThermoFisher Scientific, Waltham, MA, USA) supplemented with 10% FBS, 100 μg/mL of streptomycin, and 100 U/mL of penicillin (Gibco™, ThermoFisher Scientific, Waltham, MA, USA) and incubated at 37 °C and 10% CO_2_. Cells were passed using trypsin-EDTA (0.05%; Gibco™, ThermoFisher Scientific, Waltham, MA, USA) and the maximum passage number for the in vivo experiments was 5. Cells were tested mycoplasma-negative.

### 2.3. In Vivo Experiments

All experiments were performed in accordance with the European Directive 2010/63/EU and following the Belgian national regulation guidelines, and were accepted by the ethical committee for animal care by the Faculty of Medicine of the UCLouvain (2019/UCL/MD/004). Water and food were given ad libitum. Mice were monitored daily and were euthanized according to the following endpoints: (i) 20% body weight loss, (ii) 10% body weight loss plus clinical signs of morbidity (e.g., arched back, lack of movement, paralysis) or (iii) when the subcutaneously (SC) tumor volume reached 1500 mm^3^ (only for the re-challenge experiment).

#### 2.3.1. GL261 Mouse Models

Six-week-old female C57BL/6J mice (Charles River Laboratories, Wilmington, MA, USA) were anesthetized by intraperitoneal injection of ketamine/xylazine (100 and 13 mg/kg, respectively) and fixed on a stereotactic frame. A hole was drilled using a surgical high-speed drill (Velleman, Gavere, Belgium) and 2 µL of native DMEM containing 1.3 × 10^5^ GL261 cells were injected into the right hemisphere using an infusion syringe pump (Harvard Apparatus, Holliston, MA, USA) mounted with a Hamilton syringe (26S gauge needle). The injection coordinates were 2.1 mm lateral and 0.5 mm posterior from the bregma, and 2.6 mm deep from the outer border of the cranium. Mice were euthanized to assess the immune profile in the brain and/or in the spleen either after 21 days or at endpoints. Long-term survival was defined as 90 days post-tumor implantation.

For the re-challenge of long-term survivors, 2 × 10^6^ GL261 cells in 100 µL of PBS were injected SC in the flank. Naïve, age-matched mice were employed as controls. Tumor volumes were calculated as length × width × height (in mm^3^).

#### 2.3.2. Magnetic Resonance Imaging

The presence, volume and location of GL261 tumors in the brain were determined by magnetic resonance imaging (MRI) on days 7, 14 and 21 following tumor implantations. A supplementary MRI was conducted on day 90 for the long-term survivors. Mice were anesthetized with isoflurane mixed with air (2.5% for induction, 1.5% for maintenance). Animals were covered with a heating blanket and the temperature was monitored. A pressure pad was used to monitor the respiration rate. MRI was performed using an 11.7 T Bruker Biospec MRI system (Bruker, Billerica, MA, USA) equipped with a 1 H quadrature transmit/receive birdcage coil (21 mm inner diameter). Tumors were visualized using a T1 fast low angle shot (FLASH) sequence (repetition time = 260 ms; effective echo time = 3 ms; flip angle = 25; field of view = 20 mm × 20 mm; matrix size = 200 mm × 200 mm; resolution = 10 µm; slice thickness = 0.4 mm; acquisition time = 3 min 30 s). Gadolinium-DOTA (Dotarem^®^ 0.5 mol/mL; Guerbet, Villepinte, France) was injected intraperitoneally at a dose of 4 mmol/kg, 10 min before the acquisition [25,26]. The use of a contrast agent was necessary to confirm the presence of GL261 tumors on day 7 in order to start the treatments on day 8. Indeed, these tumors are very infiltrative and difficult to visualize at an early stage with T2-weighted MRI [27].

#### 2.3.3. Immunization

For vaccine administration, the left hind leg was shaved and 30 µL of PBS containing 10 µg of plasmid was injected into the tibial cranial muscle of C57Bl/6J mice (Charles River Laboratories, Wilmington, MA, USA). The leg was placed between 4 mm spaced plate electrodes (BTX Caliper Electrodes; VWR International), and 8 square-wave electric pulses (200 V/cm, 20 ms, 2 Hz) were delivered using a BTX Gemini System generator (VWR International, Radnor, PA, USA) [21]. A conductive gel was used to ensure electrical contact with the skin (Aquasonic 100; Parker Laboratories, Fairfield, NJ, USA). For the pTOP optimization experiment, plasmids were administered to naïve mice on days 0, 7 and 14. For therapeutic immunization, the vaccine was administered 8, 13 and 18 days following the orthotopic grafting of GL261 tumors.

Immune checkpoint blockade antibodies directed against CTLA-4 (InVivoMAb anti-mouse CTLA-4 (CD152)) and PD-1 (InVivoMAb anti-mouse PD-1 (CD279)) were purchased from Bio-connect (Huissen, The Netherlands). Mice were injected intraperitoneally with 200 μg of each antibody in 200 μL of PBS 10, 12 and 14 days after the tumor implantation.

### 2.4. IFN-γ Enzyme-Linked Immunospot Assay

For the pTOP optimization experiment, mice were sacrificed 7 days following the third dose of plasmid administration. Spleens were harvested, placed in 5 mL of cold DMEM (Gibco™, ThermoFisher Scientific, Waltham, MA, USA) and kept on ice until processing. Spleens were smashed using a 70 µm cell strainer (Greiner Bio-One). ACK (ammonium-chloride-potassium) lysing buffer (7 mL) was prepared as previously described [28] and was added for 1 min to lyse red blood cells. Splenocytes were washed with 20 mL of DMEM supplemented with 10% FBS (Gibco™, ThermoFisher Scientific, Waltham, MA, USA).

The enzyme-linked immunospot (ELISpot) assay (Mouse IFN-γ Single-Color ELISPOT) was performed according to the manufacturer’s instructions (ImmunoSpot^®^, CTL Europe GmbH, Bonn, Germany). Briefly, 3 × 10^5^ splenocytes in 100 µL of CTL-Test™ medium (ImmunoSpot^®^, CTL Europe GmbH, Bonn, Germany) were added to an anti-IFN-γ-coated 96-well plate. For stimulation, 10 ng/µL of TRP2_180–188_ peptide (SVYDFFVWL) (GenScript, Piscataway, NJ, USA) was added to the splenocytes. A cell stimulation cocktail (Invitrogen) was used as a positive control; PBS and a P815 irrelevant peptide (LPYLGWLVF) (GeneCust, Boynes, France) were used as negative controls. The plate was incubated for 2 days at 37 °C and 10% CO_2_. The plate was developed following the manufacturer’s instruction and was then sent to CTL Europe GmbH for the analysis of the spots counts on an ImmunoSpot^®^ Reader.

### 2.5. Flow Cytometry Analysis of the Immune Populations in Brains and Spleens

Brain and spleen cells were collected either 21 days after tumor implantation (for brains and spleens) or at the endpoints (brains), depending on the experiment. Cells were passed through a 70 µm cell strainer (Greiner Bio-One, Vilvoorde, Belgium), counted and washed with PBS. Cells were incubated for 10 min on ice with TruStain FcX™ blocking solution (anti-mouse CD16/32 antibody, Biolegend, San Diego, CA, USA). For MDSC detection, cells were stained with anti-CD11b (FITC rat anti-mouse CD11b; BD Bioscience, Franklin Lakes, NJ, USA) and anti-Gr1 (PE rat anti-mouse Ly-6G and Ly-6C; BD Bioscience, Franklin Lakes, NJ, USA) antibodies for 30 min at 4 °C. For CD4 and CD8 T cell detection, cells were stained with anti-CD3 (APC/Cyanine7 anti-mouse CD3; Biolegend, San Diego, CA, USA), anti-CD4 (PE rat anti-Mouse CD4; BD Bioscience, Franklin Lakes, NJ, USA) and anti-CD8 (Brilliant Violet 421™ anti-mouse CD8a; Biolegend, San Diego, CA, USA) antibodies for 30 min at 4 °C. Cells were then incubated for 30 min at RT with a permeabilization/fixation solution (eBioscience™ Foxp3/Transcription Factor Staining Buffer Set; ThermoFisher Scientific, Waltham, MA, USA) prior to incubation with anti-IFN-γ (APC anti-mouse IFN-γ; Biolegend, San Diego, CA, USA) and anti-FoxP3 (Alexa Fluor^®^ 488 rat anti-mouse Foxp3; BD Bioscience, Franklin Lakes, NJ, USA) antibodies for 1 h at 4 °C. Sample data were acquired with FACSVerse (BD bioscience, Franklin Lakes, NJ, USA)) and analyzed with FlowJo software (FlowJo, Ashland, OR, USA). The gating strategy is depicted in Figure S2.

### 2.6. Statistics

Statistical analyses were performed using the statistical software GraphPad Prism (GraphPad Software Version 9.1.2, San Diego, CA, USA). Survival was plotted using Kaplan–Meier curves and analyzed with the log-rank Mantel–Cox test. Multiple comparisons were assessed by one-way ANOVA with Dunnet’s multiple comparison tests. The *p*-values < 0.05 were considered significant.

## 3. Results

### 3.1. The Insertion Position of TRP2_180–188_ in VSV-G Influences the Immune Response

**Figure 2:**
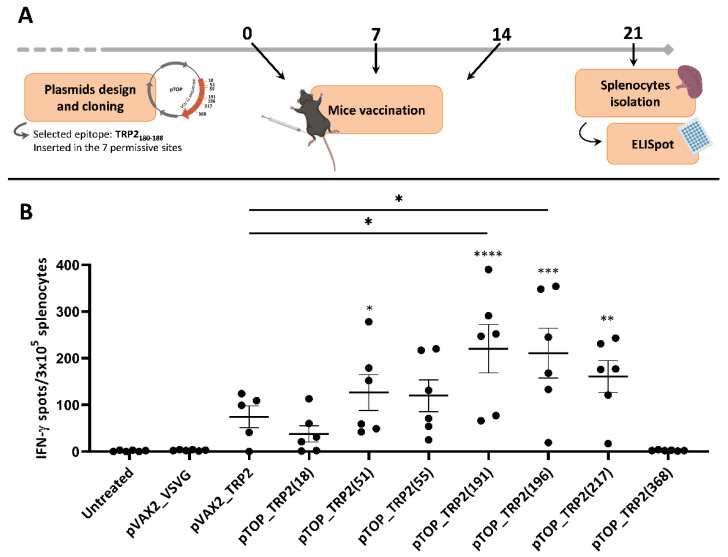
Influence of the insertion position of TRP2_180–188_ epitope in pTOP. (**A**) Schematic protocol of experiments. Timing is expressed in days. (**B**) ELISpot results assessing the production of IFN-γ by splenocytes following stimulation with TRP2_180–188_ peptide. Values are mean ± SEM; *n* = 5–6. Statistical analyses were performed using one-way ANOVA with Dunnet’s multiple comparison test (* *p* < 0.05; ** *p* < 0.01; *** *p* < 0.001; **** *p* < 0.0001, compared to the untreated group or to the specified group).

To generate a specific cellular immune response against the tyrosinase-related protein 2 (TRP2), already described as a GBM antigen [19,29], we selected the TRP2_180–188_ peptide, which is known to bind to MHC class I molecules [30]. VSV-G contains seven possible permissive sites [31]. To evaluate which insertion position in the VSV-G sequence of the DNA vaccine would be more efficient in inducing a specific and strong immune response against the selected epitope, we constructed seven pTOP plasmids with the TRP2_180–188_ sequence inserted in each of the seven permissive sites in VSV-G. We then evaluated their capacity to induce a specific IFN-γ response in naïve mice. To do so, splenocytes were collected 7 days following the third dose of DNA vaccine and were analyzed by ELISpot (Figure 2A).

Four pTOP constructs, with TRP2_180–188_ inserted in positions 51, 191, 196 and 217 (of the VSV-G protein sequence), induced a strong TRP2 specific IFN-γ response as reflected by the number of spots that are significantly higher than in the untreated group. Among these four plasmids, only two produced a number of spots significantly higher than pVAX2_TRP2: pTOP_TRP2(191) and pTOP_TRP2(196). One pTOP construct (pTOP_TRP2(368)) did not induce any response, and two (pTOP_TRP2(18) and pTOP_TRP2(55)) produced a number of spots that were not significantly higher than the controls. Splenocytes from untreated mice and from mice treated with the control plasmid pVAX2_VSVG did not induce any IFN-γ response after incubation with TRP2_180–188_. Splenocytes from mice treated with pVAX2_TRP2, the control plasmid containing the complete sequence of TRP2 without VSV-G, produced IFN-γ spots, however, their number was not significantly higher than the two other control groups (Figure 2B). For our further experiments, among the two plasmids that induced the strongest and equivalent (*p* > 0.9999) IFN-γ immune response in the ELISpot assay, we selected the pTOP_TRP2(191) vaccine that was previously used [19]. Moreover, pTOP_TRP2(191) will be referred to as pTOP.

### 3.2. Combination Therapy Induces a Potent T Cell Response in the Spleen and Increases the Activation of CD8 T Cells in the Brain

**Figure 3:**
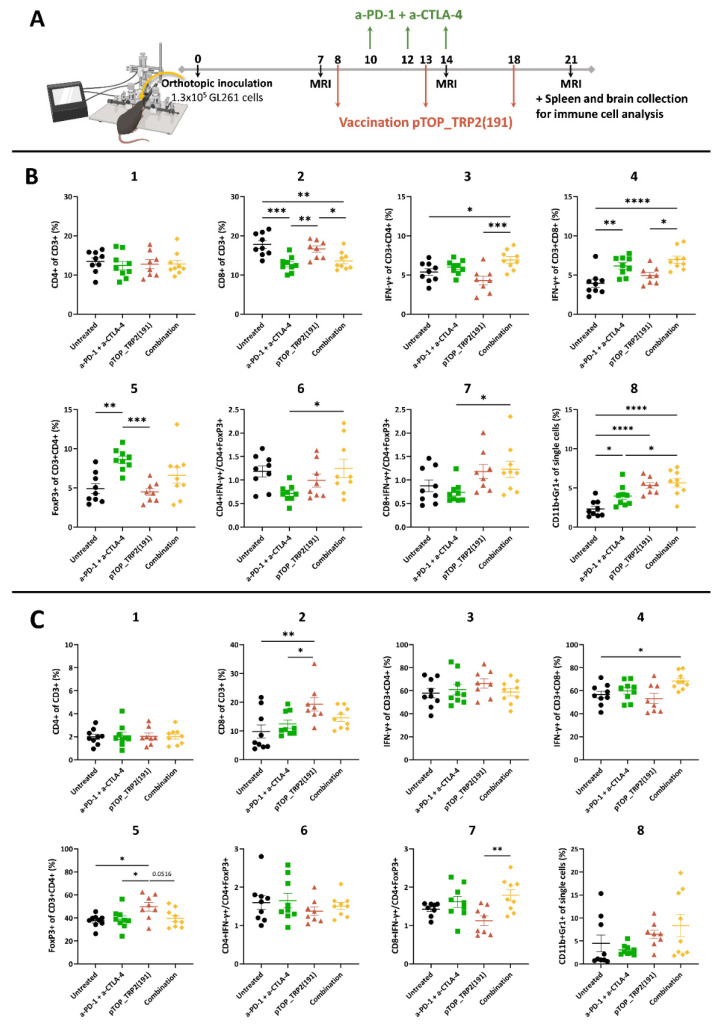
Immune cell populations analysis in the spleens and brains 21 days after tumor implantation. (**A**) Schematic protocol of experiments. Timing is expressed in days. (**B**) FACS analysis of immune cells in the spleens on day 21. (**C**) FACS analysis of immune cells in the brains on day 21. Values are mean ± SEM; *n* = 8–9. Statistical analyses were performed using one-way ANOVA with Dunnet’s multiple comparison test (* *p* < 0.05; ** *p* < 0.01; *** *p* < 0.001, **** *p* < 0.0001, compared to the specified group). Untreated: black circle; anti-PD-1 + anti-CTLA-4: green square; pTOP: red triangle; combination: yellow diamond.

We hypothesized that combining pTOP with PD-1 and CTLA-4 dual blockade would result in a stronger immune response against GBM tumors compared with either pTOP or dual blockade alone. We checked the presence of a tumor before treatment on day 7 and we followed tumoral growth by MRI on days 14 and 21 (Figure S3). To assess immunotherapeutic efficacy, GBM-bearing mice were treated with either pTOP alone, dual blockade with anti-PD-1 and anti-CTLA-4 mAbs, or with a combination of both. Mice were vaccinated with 10 µg of pTOP on days 8, 13 and 18, and the anti-PD-1 and anti-CTLA-4 mAbs were administered at a dose of 200 µg on days 10, 12 and 14. No clinical signs of adverse effects (e.g., change of body weight or behavior, rash, etc.) were observed during the treatments. Spleens and brains were then harvested on day 21 and markers of immune activation and immunosuppression were analyzed by FACS (Figure 3).

The frequency of CD8 T cells in the spleen was reduced following dual ICB and combination treatments, but not following pTOP alone. The frequency of IFN-γ-secreting CD8 T cells was significantly higher after the dual blockade and combination therapy. Only the combination was able to significantly induce an increase in IFN-γ-secreting CD4 T cells frequency in the spleen, compared to the untreated and pTOP monotherapy groups. The percentage of FoxP3+ Tregs in the spleen was increased following dual ICB. Our results demonstrated that the combination of pTOP with anti-PD-1 and anti-CTLA-4 ICBs increases the effector CD8 T cell to Treg and effector CD4 T cell to Treg ratios in the spleen compared to the other groups. Myeloid-derived suppressor cell (MDSC) markers analysis demonstrated an increase in MDSC frequency following pTOP and combination treatments in the spleen on day 21 (Figure 3B).

Immune cell infiltration in the brain was assessed on day 21. Moreover, pTOP increased the frequency of infiltrated CD8 T cells compared with the untreated and ICBs groups. The percentage of FoxP3+ Tregs was also increased following pTOP treatment compared to all other groups. Combination therapy was the only treatment leading to an increase in infiltrated IFN-γ-secreting CD8 T cells frequency compared with the untreated group. When compared to pTOP alone, the effector CD8 T cell to Treg ratio was significantly increased following combination therapy. No significant difference in the percentage of MDSCs was observed in the brain (Figure 3C).

### 3.3. Dual ICBs and Combination Therapy Increase Survival of GBM-Bearing Mice

**Figure 4:**
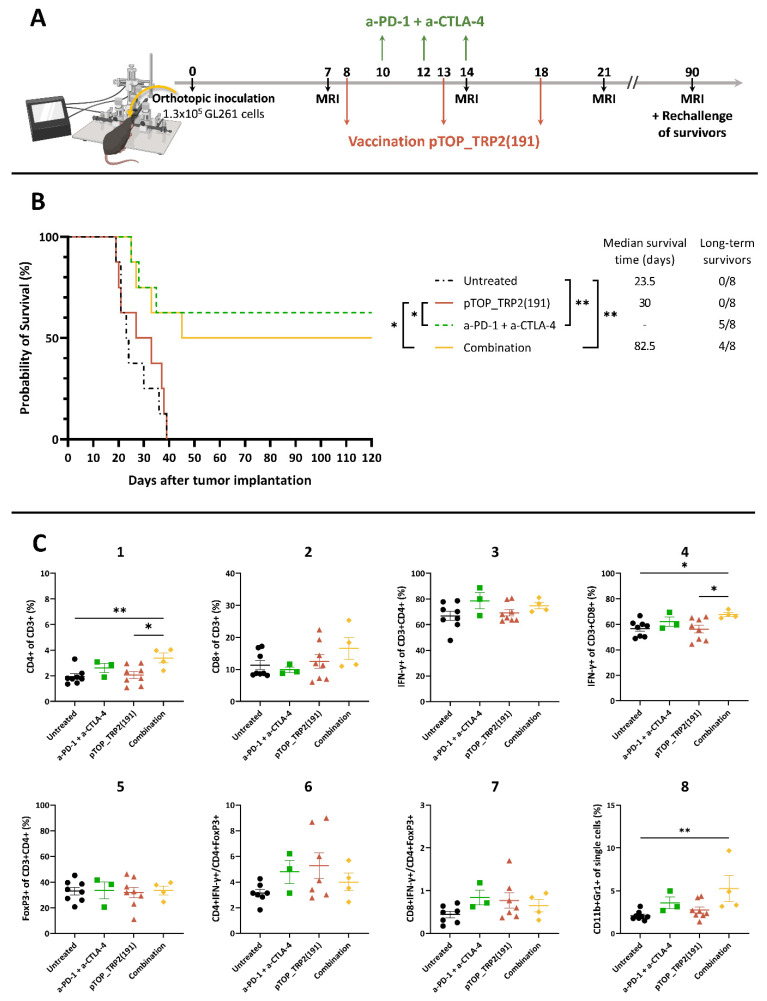
Survival of GL261-bearing mice following treatment with pTOP, anti-PD-1 and anti-CTLA-4 mAbs or the combination. (**A**) Schematic protocol of experiments. Timing is expressed in days. (**B**) Survival curves following treatment with pTOP, ICBs or combination; *n* = 8. Statistical analyses of survival curves were performed using Mantel–Cox test (* *p* < 0.05; ** *p* < 0.01). (**C**) FACS analysis of immune cells in the brain at endpoints. Values are mean ± SEM; *n* = 3–8. Statistical analyses were performed using one-way ANOVA with Dunnet’s multiple comparison test (* *p* < 0.05; ** *p* < 0.01, compared to the specified group). Untreated: black circle; anti-PD-1 + anti-CTLA-4: green square; pTOP: red triangle; combination: yellow diamond.

Given the improved T cell landscape following combination therapy, we checked whether the treatment could also improve the survival of GBM-bearing mice (Figure 4). The survival curves of the pTOP and untreated groups were not significantly different. However, the median survival time was extended following vaccination with pTOP (30 days vs. 23.5 days for the untreated group). Treatments with ICBs and with the combination significantly improved the survival and led to 5/8 and 4/8 long-term survivors, respectively. No significant difference was observed between the ICBs and combination groups (Figure 4B). MRI was performed on long-term survivors on day 90 to confirm tumor regression (Figure S3). Long-term survivors were then re-challenged with SC flank tumors and all of them rejected the tumor, suggesting that the treatments induced an immune memory response (Figure S4).

When analyzing the immune cell markers at endpoints, we observed an increase in CD4 T cells and in IFN-γ-secreting CD8 T cells frequencies in the combination group compared with the untreated and pTOP groups. No difference in the effector T cell to Treg ratios was observed. An increase in MDSC frequency was observed in the group treated with the combination compared with the untreated group (Figure 4C).

## 4. Discussion

Given the recent success of immunotherapy in aggressive cancers, these strategies are becoming increasingly explored for GBM [8]. Therapeutic cancer vaccines constitute an interesting approach to generating a strong and specific cellular immune response while inducing immune memory. However, therapeutic vaccination in monotherapy has not yet proven efficacy for GBM. We hypothesized that a combination immunotherapy, by stimulating different arms of the cancer immunity cycle, could lead to an improved outcome in the context of GBM. Recently, we developed a vaccine that showed efficacy in an orthotopic GBM model when combined with resection of the tumor. However, the efficacy in the unresected group was lower, suggesting that a combination needs to revert the cold GBM TME into hot.

Furthermore, pTOP, a cancer DNA vaccine encoding a modified VSV-G protein with defined T cell epitopes, generated a strong and specific immune response against the tumor epitope TRP2_180–188_ only when the latter was inserted in particular permissive insertion sites in the VSV-G sequence. Peptides recognized by CD8 T cells on MHC class I molecules are more often derived from the degradation of cellular protein by the proteasome. Different subtypes of proteasome exist and each of them produces different sets of peptides depending on their catalytic subunits that have different cleavage specificities (e.g., caspase-like, chymotrypsin-like or trypsin-like activities) [24]. We hypothesized that the position of the TRP2_180–188_ epitope sequence in the VSV-G protein sequence might impact the processing of the epitope by the proteasome machinery. Indeed, the amino acid sequence flanking an epitope has already been shown to impact the cleavage site preferences of the proteasome [32]. Therefore, we first analyzed the effect of the insertion position of a GBM tumor epitope in pTOP. Our results showed that positions 191 and 196 in the VSV-G protein were the more suitable positions for the insertion of the TRP2_180–188_ epitope in order to induce a strong IFN-γ immune response. We speculate that these positions are more favorable for the processing of the entire TRP2_180–188_ epitope sequence and, therefore, for the induction of a CD8 immune response. However, further analyses are required to understand deeply the role of the insertion into the pTOP cancer DNA vaccine.

Consistent with our previous results, pTOP in monotherapy was not sufficient to impact neither the immune response nor the survival in the GL261 GBM mouse model [19]. GBM is a very complex cancer to treat, due to the multifactorial immunosuppression occurring in its TME and the BBB that limits the delivery of therapeutics to the tumor site. Therefore, targeting a single arm is not enough to modify the immune landscape, as already shown in other studies where vaccines in monotherapy did not increase the immune response while combination with chemotherapy or with ICBs did [8,33,34]. Here, we combined the use of pTOP with dual blockade of PD-1 and CTLA-4. ICBs significantly improved the survival of GL261-bearing mice, as already shown in other studies [35,36,37]. Importantly, our results demonstrated that the combination therapy with pTOP and ICBs did not influence Tregs and MDSCs, but significantly improved the immune response by increasing the local infiltration of IFN-γ-secreting CD8 T cells. Although the combination therapy increased the survival compared to the untreated and pTOP groups, it was not better compared to the dual ICB therapy. These results can be explained by (i) the higher MDSC frequency observed in the combination group that could inhibit the effector T cell activity, or (ii) the high efficacy of ICBs due to the immunogenicity of GL261. Indeed, the murine GL261 model is highly immunogenic while human GBM is not [38,39]. Therefore, mice bearing GL261 could respond better to ICBs than humans, as clinical trials did not demonstrate the efficacy of using ICBs for patients facing GBM [9,40].

Following our observations, we hypothesize that GBM outcome could be improved by developing new combination strategies with treatment that will help reduce the immunosuppression, for example, by targeting Tregs or MDSCs. To ameliorate vaccine effects, targeting multiple epitopes as well as selecting GBM neoepitopes should be considered. Moreover, exploiting the proteasomal processing, as recently shown in a GBM mouse model, might be a valuable strategy [41]. Finally, switching to other mouse models that would be less immunogenic than GL261 and more closely related to human GBM should be considered. Moreover, the simultaneous use of different mouse models that react differently to ICBs should be of great interest in order to improve preclinical knowledge.

## 5. Conclusions

Targeting multiple arms against GBM is of great interest due to the intrinsic complexity of the disease. Here, we hypothesized that the combination of dual blockade of PD-1 and CTLA-4 with pTOP, a DNA vaccine encoding a CD8 epitope of TRP2 antigen, would improve the immune response and therapeutic outcome. We showed that the combination improved the immune profile of GL261 GBM-bearing mice, opening a new perspective for the treatment of GBM.

## Data Availability

Data are contained within the article and supplementary material.

## Supplementary Materials

The following supporting information can be downloaded at: https://www.mdpi.com/article/10.3390/pharmaceutics14051025/s1. Figure S1. Protein sequence of VSV-G. The possible insertion sites are colored in blue; Figure S2. FACS gating strategy for T cell analysis; Figure S3. Follow-up of tumoral growth by MRI. A representative mouse was selected for each group; Figure S4. Rechallenge of long-term survivors. (A) Comparison of tumor volumes between the three groups. Values are mean ± SEM; *n* = 3–6. (B) Evolution of tumor volumes of each mouse from the untreated group; *n* = 6. (C) Survival curves following rechallenge of long-term survivors from the group treated with ICBs and with the combination; *n* = 3–6. Statistical analyses were performed using Mantel–Cox test (** *p* < 0.01).

## References

[1] Stupp R., Brada M., van den Bent M.J., Tonn J.-C., Pentheroudakis G. (2014). High-Grade Glioma: ESMO Clinical Practice Guidelines for Diagnosis, Treatment and Follow-Up. Ann. Oncol..

[2] Lukas R.V., Wainwright D.A., Ladomersky E., Sachdev S., Sonabend A.M., Stupp R. (2019). Newly Diagnosed Glioblastoma: A Review on Clinical Management. Oncol. Williston Park N.

[3] Lesueur P., Lequesne J., Grellard J.-M., Dugué A., Coquan E., Brachet P.-E., Geffrelot J., Kao W., Emery E., Berro D.H. (2019). Phase I/IIa Study of Concomitant Radiotherapy with Olaparib and Temozolomide in Unresectable or Partially Resectable Glioblastoma: OLA-TMZ-RTE-01 Trial Protocol. BMC Cancer.

[4] Yabroff K.R., Harlan L., Zeruto C., Abrams J., Mann B. (2012). Patterns of Care and Survival for Patients with Glioblastoma Multiforme Diagnosed during 2006. Neuro-oncology.

[5] Cameron F., Whiteside G., Perry C. (2011). Ipilimumab: First Global Approval. Drugs.

[6] Hazarika M., Chuk M.K., Theoret M.R., Mushti S., He K., Weis S.L., Putman A.H., Helms W.S., Cao X., Li H. (2017). U.S. FDA Approval Summary: Nivolumab for Treatment of Unresectable or Metastatic Melanoma Following Progression on Ipilimumab. Clin. Cancer Res..

[7] Saxena M., van der Burg S.H., Melief C.J.M., Bhardwaj N. (2021). Therapeutic Cancer Vaccines. Nat. Rev. Cancer.

[8] Bausart M., Préat V., Malfanti A. (2022). Immunotherapy for Glioblastoma: The Promise of Combination Strategies. J. Exp. Clin. Cancer Res..

[9] Reardon D.A., Brandes A.A., Omuro A., Mulholland P., Lim M., Wick A., Baehring J., Ahluwalia M.S., Roth P., Bähr O. (2020). Effect of Nivolumab vs. Bevacizumab in Patients with Recurrent Glioblastoma: The Checkmate 143 Phase 3 Randomized Clinical Trial. JAMA Oncol..

[10] Weller M., Butowski N., Tran D.D., Recht L.D., Lim M., Hirte H., Ashby L., Mechtler L., Goldlust S.A., Iwamoto F. (2017). Rindopepimut with Temozolomide for Patients with Newly Diagnosed, EGFRvIII-Expressing Glioblastoma (ACT IV): A Randomised, Double-Blind, International Phase 3 Trial. Lancet Oncol..

[11] Liau L.M., Ashkan K., Tran D.D., Campian J.L., Trusheim J.E., Cobbs C.S., Heth J.A., Salacz M., Taylor S., D’Andre S.D. (2018). First Results on Survival from a Large Phase 3 Clinical Trial of an Autologous Dendritic Cell Vaccine in Newly Diagnosed Glioblastoma. J. Transl. Med..

[12] Hatoum A., Mohammed R., Zakieh O. (2019). The Unique Invasiveness of Glioblastoma and Possible Drug Targets on Extracellular Matrix. Cancer Manag. Res..

[13] Karmur B.S., Philteos J., Abbasian A., Zacharia B.E., Lipsman N., Levin V., Grossman S., Mansouri A. (2020). Blood-Brain Barrier Disruption in Neuro-Oncology: Strategies, Failures, and Challenges to Overcome. Front. Oncol..

[14] Pombo Antunes A.R., Scheyltjens I., Duerinck J., Neyns B., Movahedi K., Van Ginderachter J.A. (2020). Understanding the Glioblastoma Immune Microenvironment as Basis for the Development of New Immunotherapeutic Strategies. eLife.

[15] Garg A.D., Vandenberk L., Van Woensel M., Belmans J., Schaaf M., Boon L., De Vleeschouwer S., Agostinis P. (2017). Preclinical Efficacy of Immune-Checkpoint Monotherapy Does Not Recapitulate Corresponding Biomarkers-Based Clinical Predictions in Glioblastoma. OncoImmunology.

[16] Skaga E., Kulesskiy E., Fayzullin A., Sandberg C.J., Potdar S., Kyttälä A., Langmoen I.A., Laakso A., Gaál-Paavola E., Perola M. (2019). Intertumoral Heterogeneity in Patient-Specific Drug Sensitivities in Treatment-Naïve Glioblastoma. BMC Cancer.

[17] Bao Z., Wang Y., Wang Q., Fang S., Shan X., Wang J., Jiang T. (2021). Intratumor Heterogeneity, Microenvironment, and Mechanisms of Drug Resistance in Glioma Recurrence and Evolution. Front. Med..

[18] Grossman S.A., Ye X., Piantadosi S., Desideri S., Nabors L.B., Rosenfeld M., Fisher J., NABTT CNS Consortium (2010). Survival of Patients with Newly Diagnosed Glioblastoma Treated with Radiation and Temozolomide in Research Studies in the United States. Clin. Cancer Res..

[19] Lopes A., Bastiancich C., Bausart M., Ligot S., Lambricht L., Vanvarenberg K., Ucakar B., Gallez B., Préat V., Vandermeulen G. (2021). New Generation of DNA-Based Immunotherapy Induces a Potent Immune Response and Increases the Survival in Different Tumor Models. J. Immunother. Cancer.

[20] Lambricht L., Lopes A., Kos S., Sersa G., Préat V., Vandermeulen G. (2016). Clinical Potential of Electroporation for Gene Therapy and DNA Vaccine Delivery. Expert Opin. Drug Deliv..

[21] Vandermeulen G., Vanvarenberg K., De Beuckelaer A., De Koker S., Lambricht L., Uyttenhove C., Reschner A., Vanderplasschen A., Grooten J., Préat V. (2015). The Site of Administration Influences Both the Type and the Magnitude of the Immune Response Induced by DNA Vaccine Electroporation. Vaccine.

[22] Taggart D., Andreou T., Scott K.J., Williams J., Rippaus N., Brownlie R.J., Ilett E.J., Salmond R.J., Melcher A., Lorger M. (2018). Anti–PD-1/Anti–CTLA-4 Efficacy in Melanoma Brain Metastases Depends on Extracranial Disease and Augmentation of CD8 T Cell Trafficking. Proc. Natl. Acad. Sci. USA.

[23] Nayyar N., Singh M., Stocking J., Brehm M., Brastianos P. (2020). 62. Presence of Extracranial Tumors Influences Response to Immune Checkpoint Inhibitors in a Pre-Clinical Model of Melanoma Brain Metastasis. Neuro-Oncol. Adv..

[24] Vigneron N., Van den Eynde B.J. (2012). Proteasome Subtypes and the Processing of Tumor Antigens: Increasing Antigenic Diversity. Curr. Opin. Immunol..

[25] Howles G.P., Bing K.F., Qi Y., Rosenzweig S.J., Nightingale K.R., Johnson G.A. (2010). Contrast-Enhanced In Vivo Magnetic Resonance Microscopy of the Mouse Brain Enabled by Noninvasive Opening of the Blood-Brain Barrier with Ultrasound. Magn. Reson. Med..

[26] Moreno H., Hua F., Brown T., Small S. (2006). Longitudinal Mapping of Mouse Cerebral Blood Volume with MRI. NMR Biomed..

[27] McKelvey K.J., Hudson A.L., Prasanna Kumar R., Wilmott J.S., Attrill G.H., Long G.V., Scolyer R.A., Clarke S.J., Wheeler H.R., Diakos C.I. (2020). Temporal and Spatial Modulation of the Tumor and Systemic Immune Response in the Murine Gl261 Glioma Model. PLoS ONE.

[28] Brown W.E., Hu J.C., Athanasiou K.A. (2016). Ammonium–Chloride–Potassium Lysing Buffer Treatment of Fully Differentiated Cells Increases Cell Purity and Resulting Neotissue Functional Properties. Tissue Eng. Part C Methods.

[29] Blaszczyk-Thurin M., Shen C.T., Ertl H.C. (2003). A DNA Vaccine Expressing Tyrosinase-Related Protein-2 Induces T-Cell-Mediated Protection against Mouse Glioblastoma. Cancer Gene Ther..

[30] Parkhurst M.R., Fitzgerald E.B., Southwood S., Sette A., Rosenberg S.A., Kawakami Y. (1998). Identification of a Shared HLA-A*0201-Restricted T-Cell Epitope from the Melanoma Antigen Tyrosinase-Related Protein 2 (TRP2). Cancer Res..

[31] Ammayappan A., Peng K.-W., Russell S.J. (2013). Characteristics of Oncolytic Vesicular Stomatitis Virus Displaying Tumor-Targeting Ligands. J. Virol..

[32] Toes R.E.M., Nussbaum A.K., Degermann S., Schirle M., Emmerich N.P.N., Kraft M., Laplace C., Zwinderman A., Dick T.P., Müller J. (2001). Discrete Cleavage Motifs of Constitutive and Immunoproteasomes Revealed by Quantitative Analysis of Cleavage Products. J. Exp. Med..

[33] Fritzell S., Sandén E., Eberstål S., Visse E., Darabi A., Siesjö P. (2013). Intratumoral Temozolomide Synergizes with Immunotherapy in a T Cell-Dependent Fashion. Cancer Immunol. Immunother..

[34] Jahan N., Talat H., Alonso A., Saha D., Curry W.T. (2019). Triple Combination Immunotherapy with GVAX, Anti-PD-1 Monoclonal Antibody, and Agonist Anti-OX40 Monoclonal Antibody Is Highly Effective against Murine Intracranial Glioma. OncoImmunology.

[35] Genoud V., Marinari E., Nikolaev S.I., Castle J.C., Bukur V., Dietrich P.-Y., Okada H., Walker P.R. (2018). Responsiveness to Anti-PD-1 and Anti-CTLA-4 Immune Checkpoint Blockade in SB28 and GL261 Mouse Glioma Models. OncoImmunology.

[36] Saha D., Martuza R.L., Rabkin S.D. (2017). Macrophage Polarization Contributes to Glioblastoma Eradication by Combination Immunovirotherapy and Immune Checkpoint Blockade. Cancer Cell.

[37] Wainwright D.A., Chang A.L., Dey M., Balyasnikova I.V., Kim C.K., Tobias A., Cheng Y., Kim J.W., Qiao J., Zhang L. (2014). Durable Therapeutic Efficacy Utilizing Combinatorial Blockade against IDO, CTLA-4, and PD-L1 in Mice with Brain Tumors. Clin. Cancer Res..

[38] Haddad A.F., Young J.S., Amara D., Berger M.S., Raleigh D.R., Aghi M.K., Butowski N.A. (2021). Mouse Models of Glioblastoma for the Evaluation of Novel Therapeutic Strategies. Neuro-Oncol. Adv..

[39] Xu M., Yao Y., Hua W., Wu Z., Zhong P., Mao Y., Zhou L., Luo F., Chu Y. (2014). Mouse Glioma Immunotherapy Mediated by A2B5 + GL261 Cell Lysate-Pulsed Dendritic Cells. J. Neurooncol..

[40] Chamberlain M.C., Kim B.T. (2017). Nivolumab for Patients with Recurrent Glioblastoma Progressing on Bevacizumab: A Retrospective Case Series. J. Neurooncol..

[41] Fidanza M., Gupta P., Sayana A., Shanker V., Pahlke S.-M., Vu B., Krantz F., Azameera A., Wong N., Anne N. (2021). Enhancing Proteasomal Processing Improves Survival for a Peptide Vaccine Used to Treat Glioblastoma. Sci. Transl. Med..

