# Supplementary material for: Combination of DNA Vaccine and Immune Checkpoint Blockades Improves the Immune Response in an Orthotopic Unresectable Glioblastoma Model
Source: Pharmaceutics. 2022 May 10;14(5):1025. doi: 10.3390/pharmaceutics14051025 (PMC9145362; doi:10.3390/pharmaceutics14051025)
Supplement: Supplementary file 1 [file pharmaceutics-14-01025-s001.zip › pharmaceutics-1674634-supplementary.pdf]

18 ↓

1 MKCLLYLAFL FIGVNC**KF**TI VFPHNQKGNW KNVPSNYHYC PSSSDLNWH**N**

51 ↓ 55 ↓

51 **D**LI**GT**AIQVK MPKSHKAIQA DGWMCHASKW VTTCDFRWYG PKYITQSIRS

101 FTPSVEQCKE SIEQTKQGTW LNPGFPPQSC GYATVTDAEA VIVQVTPHHV

191 ↓ 196 ↓

151 LVDEYTGEWV DSQFINGKCS NYICPTVHNS TTWHSDYKV**K** **G**LCD**SN**LISM

217 ↓

201 DITFFSEDGE LSSLG**KE**GTG FRSNYFAYET GGKACKMQYC KHWGVRLPSG

251 VWFEMADKDL FAAARFPECP EGSSISAPSQ TSVDVSLIQD VERILDYSLC

301 QETWSKIRAG LPISPVDLSY LAPKNPGTGP AFTIINGTLK YFETRYIRVD

368 ↓

351 IAAPILSRMV GMISGT**TT**ER ELWDDWAPYE DVEIGPNGVL RTSSGYKFPL

401 YMIGHGMLDS DLHLSSKAQV FEHPHIQDAA SQLPDDESLF FGDTGLSKNP

451 IELVEGWFSS WKSSIASFFF IIGLIIGLFL VLRVGIHLCI KLKHTKKRQI

501 YTDIEMNRLG K*

Figure S1. Protein sequence of VSV-G. The possible insertion sites are colored in blue.

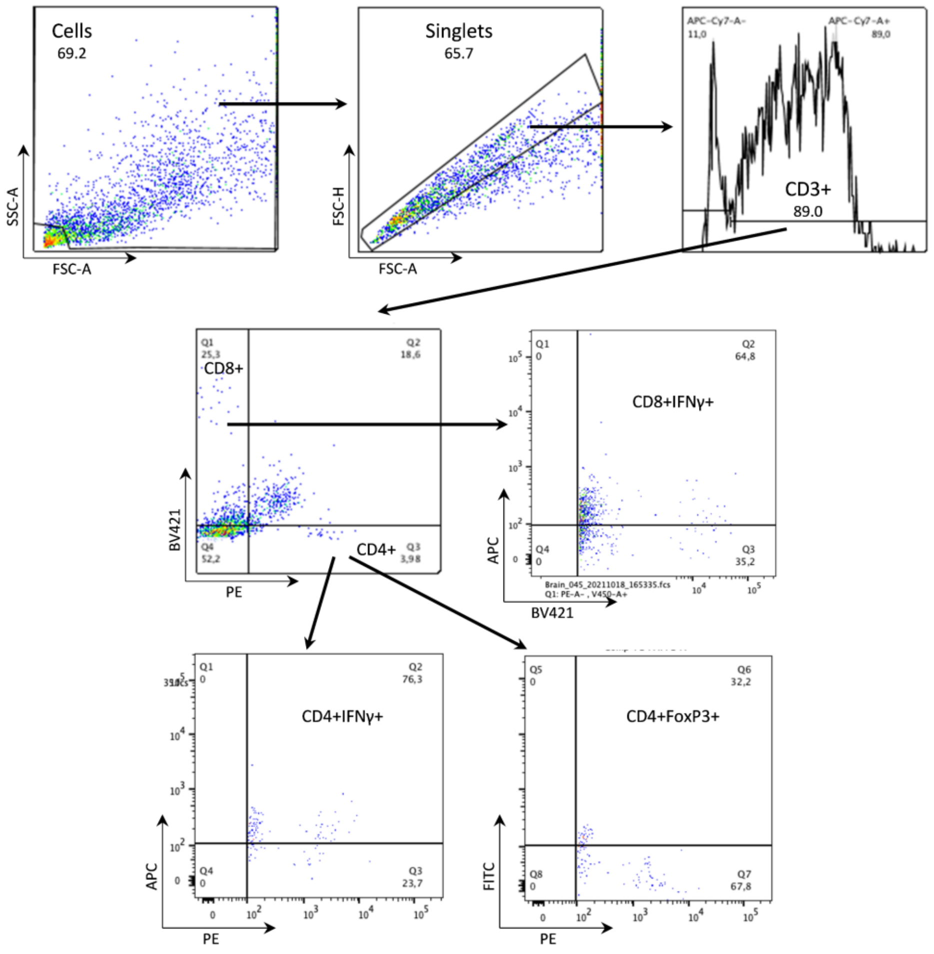

Figure S2. FACS gating strategy for T cell analysis.

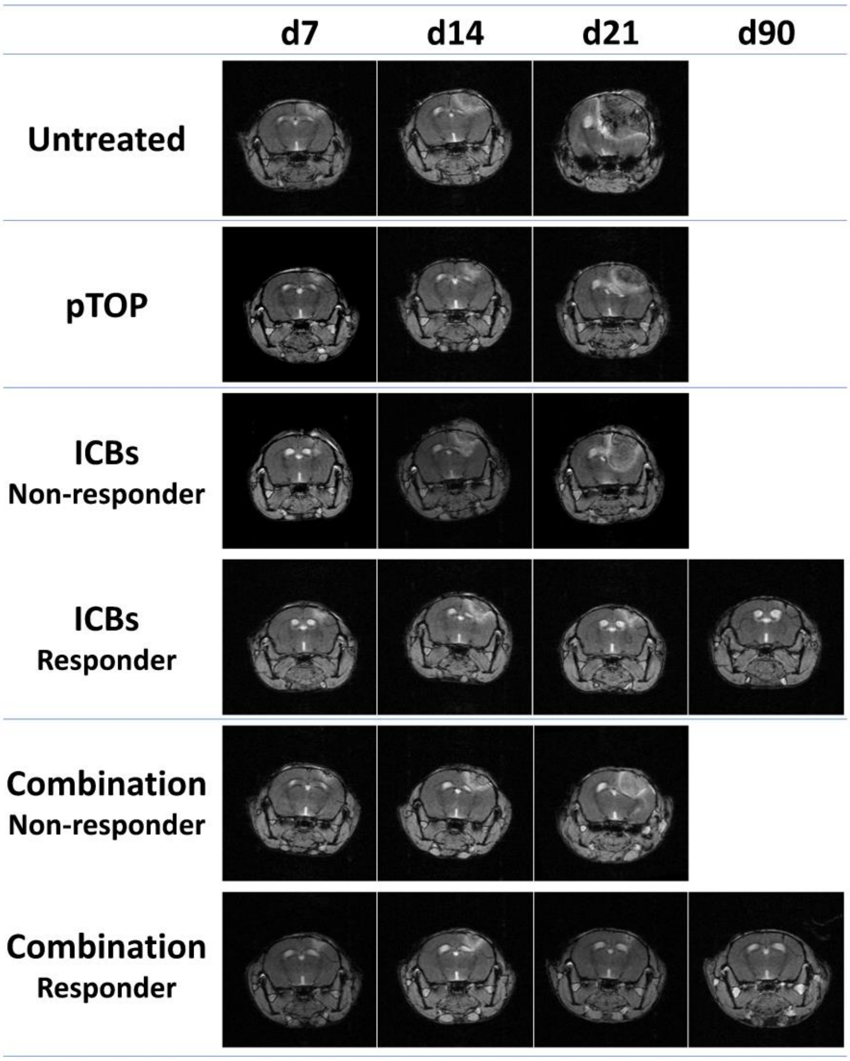

Figure S3. Follow-up of tumoral growth by MRI. A representative mouse was selected for each group.

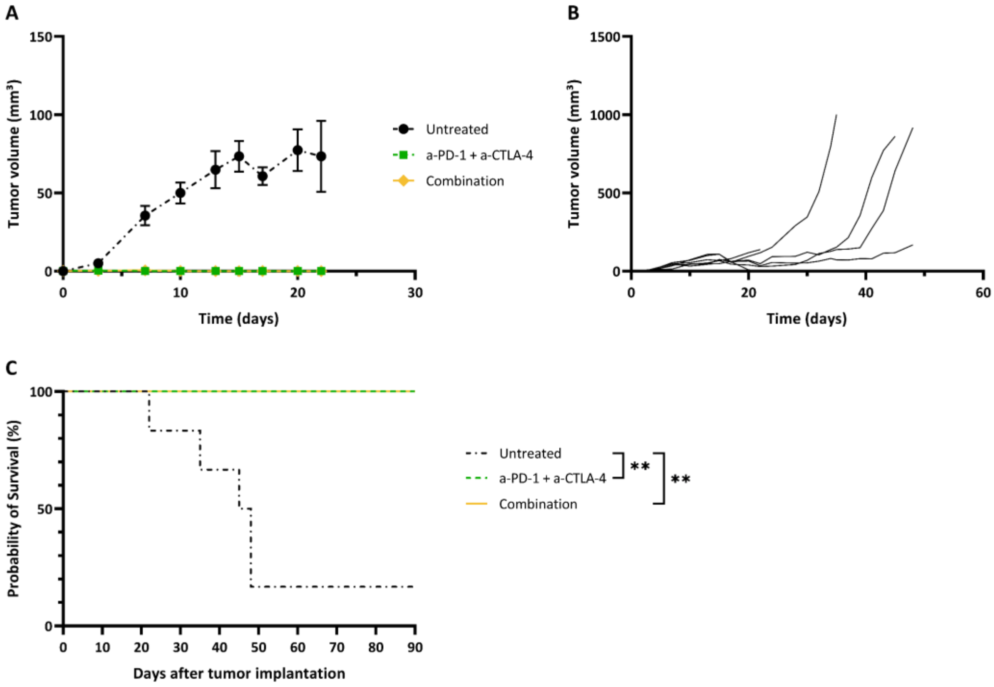

Figure S4. Rechallenge of long-term survivors. (A) Comparison of tumor volumes between the three groups. Values are mean ± SEM; n = 3-6. (B) Evolution of tumor volumes of each mouse from the untreated group; n = 6. (C) Survival curves following rechallenge of long-term survivors from the group treated with ICBs and with the combination; n = 3-6. Statistical analyses were performed using Mantel-Cox test (** P < 0.01).
